# Cost effectiveness analysis for commonly used human cell and tissue products in the management of diabetic foot ulcers

**DOI:** 10.1002/hsr2.1991

**Published:** 2024-03-22

**Authors:** Leo M. Nherera, Jaideep Banerjee

**Affiliations:** ^1^ Global Market Access; Smith + Nephew 5600 Clearfork Main St Fort Worth 76107 TX USA; ^2^ Medical Science Liaisons and Clinical Strategy Global Clinical Affairs, R&D 5600 Clearfork Main St Fort Worth 76107 TX USA

**Keywords:** cellular and/or tissue‐based products, CAMPs, cost‐effectiveness, cost‐utility, Diabetic foot ulcers

## Abstract

**Background and Aims:**

This study considers the cost‐effectiveness of commonly used cellular, acellular, and matrix‑like products (CAMPs) of human origin also known as human cell and tissue products (HCT/Ps) in the management of diabetic foot ulcers.

**Methods:**

We developed a 1‐year economic model assessing six CAMPs [cryopreserved placental membrane with viable cells (vCPM), bioengineered bilayered living cellular construct (BLCC), human fibroblast dermal substitute (hFDS), dehydrated human amnion chorion membrane (dHACM), hypothermically stored amniotic membrane (HSAM) and human amnion membrane allograft (HAMA) which had randomized controlled trial evidence compared with standard of care (SoC). CAMPs were compared indirectly and ranked in order of cost‐effectiveness using SoC as the baseline, from a CMS/Medicare's perspective.

**Results:**

The mean cost, healed wounds (hw) and QALYs per patient for vCPM is $10,907 (0.914 hw, 0.783 QALYs), for HAMA $11,470 (0.903 hw, 0.780 QALYs), for dHACM $15,862 (0.828 hw, 0.764 QALYs), for BLCC $18,430 (0.816 hw, 0.763 QALYs), for hFDS $19,498 (0.775 hw, 0.757 QALYs), for SoC $19,862 (0.601 hw, 0.732 QALYs) and $24, 214 (0.829, 0.763 QALYs) for HSAM respectively. Over 1 year, vCPM results in cheaper costs overall and better clinical outcomes compared to other CAMPs. Following probabilistic sensitivity analysis, vCPM has a 60%, HAMA 40% probability of being cost‐effective then dHACM, hFDS, BLCC, and lastly HSAM using a $100,000/healed wound or QALY threshold.

**Conclusions:**

All CAMPs were shown to be cost‐effective when compared to SoC in managing DFUs. However, vCPM appears to be the most cost‐effective CAMP over the modelled 52 weeks followed by HAMA, dHACM, hFDS, BLCC, and HSAM. We urge caution in interpreting the results because we currently lack head‐to‐head evidence comparing all these CAMPs and therefore suggest that this analysis be updated when more direct evidence of CAMPs becomes available.

## Key messages

- Human cell and tissue products (CAMPs) are cost‐effective when compared to standard of care alone in managing diabetic foot ulcers.
- Using an indirect comparison, vCPM was found to be the least costly strategy over 1 year with the most clinical benefits measured in healing rates and QALYs.

## 1 INTRODUCTION

The number of people with diabetes in the US population in 2018 was estimated to be 10.5%.^1^, ^2^ Diabetes imposes both emotional and financial burden on the patients,^3^ health insurance providers, and government‐funded healthcare organisations.^4^ In 2017, the cost of treating diabetes in the United States was estimated at $327 billion.^2^ About a quarter of people with diabetes develop diabetic foot ulcers (DFU), which adds to the financial burden as the cost of treating DFUs is estimated to be $0.6–4.5 billion, rising to $6–$18.7 billion when the cost of managing infection is included in total Medicare spending.^5^ Managing patients with DFUs costs twice as much compared to managing patients without diabetes.^6^

The goal of DFU management is to reduce the risk of ulcer complications and restore a patient's ‘pre‐ulcer’ health status.^7^ This is usually achieved by using standard of care (SoC) or good wound care. SoC generally refers to the best available treatment and consists mainly of debridement, infection control, off‐loading, and maintenance of a moist wound environment.^8^, ^9^ Despite the use of SoC, DFUs may become chronic and or get infected, which may result in further hospital admissions and possibly a lower limb amputation.^10^

Advanced wound care products such as skin substitutes otherwise known as cellular, acellular, and matrix‑like products (CAMPs) can be used adjunctively with standard care in the management of DFUs. As per a consensus document recently developed, CAMPs are defined as “a broad category of biomaterials, synthetic materials or biosynthetic matrices that support repair or regeneration of injured tissues through various mechanisms of action”.^11^ A recent report by the Agency for Healthcare Research and Quality (AHRQ) found that there are more than 70 approved skin substitutes in the management of DFUs.^12^ Skin substitutes have recently been evaluated in four different systematic reviews and meta‐analyses^13^, ^14^, ^15^, ^16^ and have been shown to be effective with favourable healing rates and reduced time to healing within 12–16 weeks. The availability of numerous options of skin substitutes has necessitated the need for comparative clinical and cost‐effectiveness studies of some of the most used skin substitutes.^4^

According to the Institute for Healthcare Improvement Triple Aim initiative, providers of care are expected to continue providing quality healthcare while reducing the costs of healthcare provision.^17^ It is against this backdrop that healthcare providers are beginning to incorporate cost‐effectiveness in their treatment decisions. One way of achieving this aim is by conducting a healthcare economic analysis. The comparative health‐economic impact of CAMPs compared to standard care has been established for individual CAMPs.^7^, ^8^, ^18^, ^19^, ^20^ However, the comparative cost‐effectiveness of CAMPs has not been done due to the heterogeneity of trial populations^18^ and the lack of head‐to‐head comparisons of the CAMPs. Nonetheless, the commonly used CAMPs have randomized controlled trial data compared to SoC. Using these published randomized controlled trials (RCTs), our study evaluated the cost‐effectiveness of CAMPs in an outpatient setting as adjunctive treatments to SoC. CAMPs can be of human or nonhuman origin, and whether they have differences in clinical efficacies is still being debated. In this paper, we have only included the CAMPs of human origin to avoid unknown variability between human‐origin and nonhuman origin products. We intend to extend this work to include CAMPs of nonhuman origin in a follow‐up study. The analysis will rank the CAMPs in order of cost‐effectiveness using SoC as the base comparator from the perspective of CMS/Medicare's which is the largest payor in the United States of America.

## 2 MATERIALS AND METHODS

**Figure 1:**
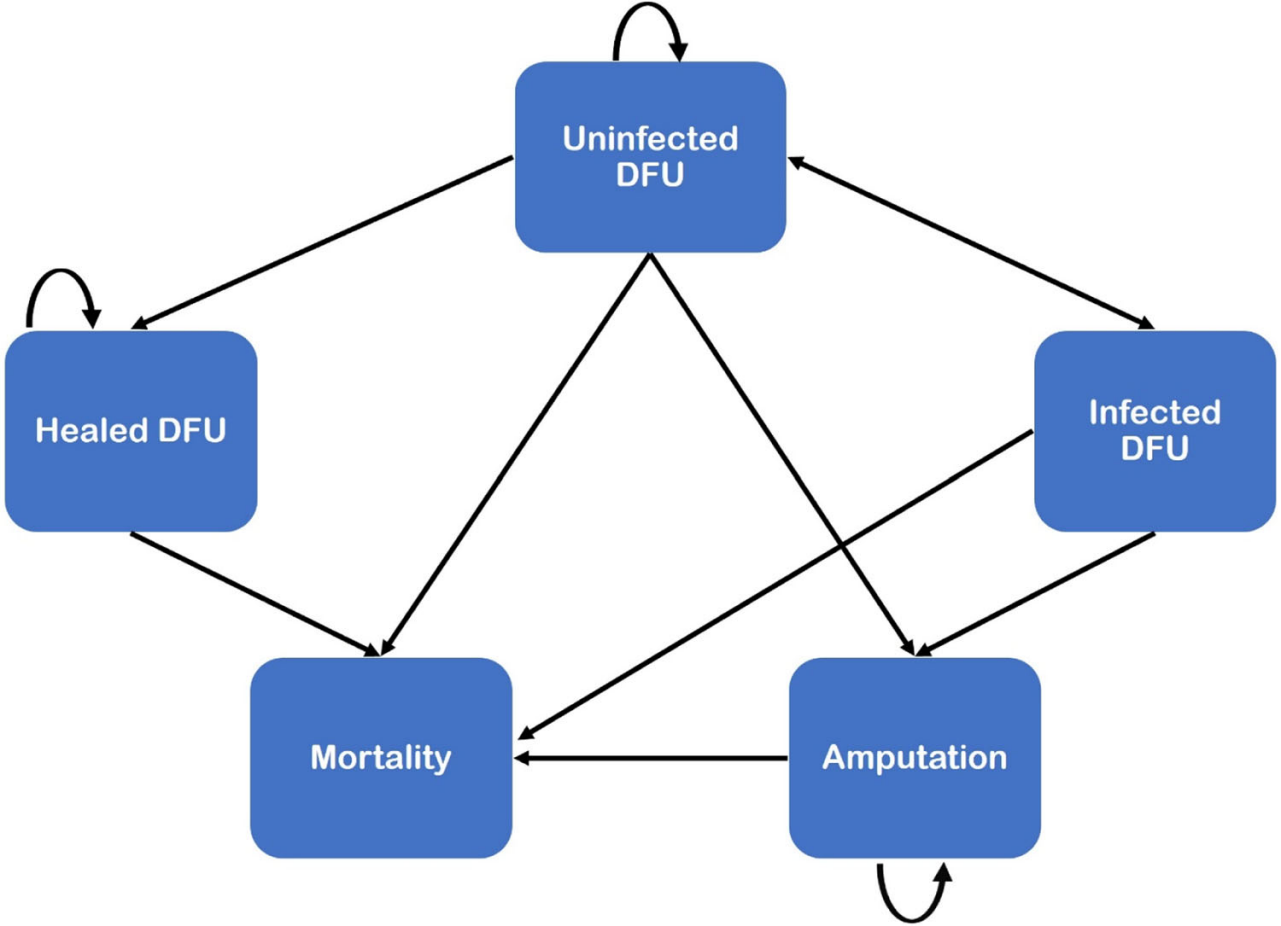
Model structure for CAMPs in managing diabetic foot ulcers. *The rounded arrow means it is a post state for instance post amputation state, post healed DFU state, and post uninfected DFU state. The arrows represent a transition from one health state to another, for instance, patients can move to the mortality state from any of the health states*.

The patient population consist of patients with uninfected DFUs at baseline. Furthermore, most commercially available CAMPs are contraindicated or not recommended to be used in active infection.^21^, ^22^ The DFU ought to have been chronic, defined as an ulcer not reducing in size by at least 50% following 4 weeks of SoC treatment. We then developed a Markov model (Figure 1) with a 1‐year time horizon from a US payer's perspective, using Microsoft® Excel (Microsoft Corporation Redmond, WA). The model was developed per recommended guidelines for conducting economic evaluations.^23^, ^24^

The Markov model allows for the simulation of foot ulcer‐related complications over 52 weeks. The model consists of eight health states including unhealed ulcer, post‐unhealed ulcer, healed ulcer, post‐healed ulcer, amputation, postamputation, infected health state, and death which is the absorbing state. A patient is expected to be in one health state at a time but can transition between health states at discrete time periods called cycles. Our model used a weekly cycle in line with other published studies, a period that is deemed sufficient to observe healing or resolution of infections.^8^, ^25^, ^26^

All people enter the model in the unhealed health state. In each model cycle, people with unhealed ulcers can heal, remain unhealed, die, and get infection. The healed ulcer represents ulcers that healed after treatment when the ulcer achieved 100% re‐epithelialization without evidence of bleeding or drainage.^25^ People with healed ulcers may remain healed or were assumed to die in each cycle. The infected ulcer state stood for a DFU that got infected. Patients from this state could move to the unhealed ulcer state, get an amputation, or die. Amputations were assumed to occur from the infected DFU state, and people in this health state could transition to the postamputation health state or die. Amputees were assumed to be at an increased risk of mortality due to the procedure. In each model cycle, they were assumed to either remain in this health state or die.

Patients are always exposed to the risk of death from any health state, and death is an absorbing state, meaning that once a patient enters this state, they cannot come out of it. Transition probabilities were derived from the published literature.

## 3 INTERVENTIONS

The model compared SoC or good wound care practice with commonly used CAMPs in the management of DFUs. This study focused on CAMPs which had randomized control data with a screening period to ensure non‐healing DFUs were enrolled in the studies. The commonly used CAMPs were cryopreserved placental membrane with viable cells (vCPM) (Grafix®, Osiris Therapeutics, Inc., Columbia, MD, USA), bioengineered bilayered living cellular construct (BLCC) (Apligraf®, Organogenesis Inc., Canton, MA), human fibroblast dermal substitute (hFDS; Dermagraft®, Organogenesis Inc., Canton, MA), dehydrated human amnion chorion membrane (dHACM; Epifix®, MiMedx Group Inc., Marietta, GA), hypothermically stored amniotic membrane (HSAM; Affinity Organogenesis Inc., Canton, MA), and human amnion membrane allograft (HAMA; AmnioBand® MTF Biologics, Edison NJ).

### 3.1 Transition probabilities

Baseline clinical data used in the model are presented in Table 1 while the treatment effect of the CAMPs is presented in Table 2, and these data were obtained from published literature. Data without CAMPs or SoC treatment for transitioning from an uncomplicated DFU to healing and DFU to infection was taken from a systematic review and meta‐analysis of CAMPs use in chronic DFUs conducted by Su et al. 2020.^13^ Su et al.^13^ included 7 randomized controlled trials in their meta‐analysis and the inclusion/exclusion criteria for these studies were comparable. We identified additional studies and performed an additional meta‐analysis. For the additional meta‐analysis we assessed the studies for statistical heterogeneity and when it was detected, we used the random effects model to assess treatment effect. Three studies^29^, ^30^, ^31^ reported data on infection compared to standard of care. We combined these data in a meta‐analysis and estimated the overall effect of CAMPs on infection to be RR 0.49 (95% CI 0.28 to 0.85, *p* = 0.01). We did not find evidence suggesting any difference between the CAMPs with regard to infection and therefore assumed that the reduction in infection rates was similar after treatment with any of the CAMPs. Event rates in the SoC arms were extracted and supplied the data without CAMPs treatment. Transitions following the infected health state (healing, amputation, and mortality) were obtained from a multicenter, prospective, observational study conducted in the UK.^27^ The study considered outcomes in patients with clinically infected diabetic foot ulcers over 12 months from diabetic foot ulcer clinics. Data on mortality following a DFU and amputation was obtained from a review study by Armstrong et al. 2021.^28^ Compared to standard of care, CAMPs were found to reduce amputation rates by 73% (RR 0.27 95% CI 0.17 to 0.44, *p* < 0.001). The model assumed mortality to be the same between the various comparators as we did not find any evidence suggesting differences in mortality between the interventions. All‐cause mortality was obtained from the US life tables.^32^

**Table 1:** Baseline clinical data used in the model (standard of care data).

Parameter	Mean	LC	UC	Weekly transition probabilities	Source
Healing	33.00%	28.39%	37.61%	2.75%	Meta‐analysis
Amputation	17.40%	13.10%	21.70%	0.33%	Ndosi^27^
DFU to healing SC	38.29%	31.89%	44.68%	3.19%	Su^13^
DFU to infection	19.61%	14.16%	25.06%	1.63%	Su^13^
DFU to amputation	6.81%	5.34%	8.28%	0.57%	Armstrong^28^
DFU no infection mortality	6.10%	1.41%	10.79%	0.12%	Armstrong^28^
DFU mortality amputation	10.28%	4.33%	16.23%	0.20%	Armstrong^28^
Mortality following infection	15.05%	11.00%	19.10%	0.29%	Ndosi^27^
**All‐cause mortality**					
	Mean annual	Weekly	Source		
All	4.87%	0.09%	US Life Tables		
55	0.22%	0.00%			
55‐64	0.89%	0.02%			
65‐74	1.86%	0.04%			
75	13.85%	0.27%			

LC, lower 95% confidence interval, UC, upper 95% confidence interval, DFU, diabetic foot ulcer, SC, standard care.

**Table 2:** Treatment effect (Risk Ratio for wound closure).

CAMPs	Mean	LC	UC	Model Value	Source
vCPM	2.91	1.61	5.26	2.91	Lavery^29^
dHACM	1.63	1.32	2.02	1.63	Zelen 2016d, Tettelbach 2019a
hFDS	1.310	1.010	1.690	1.31	Marston,^30^ Naughton 1997, T‐Fossuo 2019
BLCC	1.550	1.230	1.940	1.55	Edmonds, Zelen 2016c, Veves 2001
HSAM	1.64	1.01	2.68	1.64	Serena 2019
HAMA	2.62	1.64	4.16	2.62	DiDomenico^31^
Amputations	0.27	0.17	0.44	0.27	Armstrong^28^
Infection	0.49	0.28	0.85	0.49	Lavery,^29^ DiDomenico,^31^ Marston^30^

LC, lower 95% confidence interval, UC, upper 95% confidence interval, vCPM; Cryopreserved placental membrane with viable cells; BLCC, bioengineered bilayered living cellular construct; hFDS, Human fibroblast dermal substitute; dHACM, Dehydrated human amnion chorion membrane; HSAM, hypothermically stored amniotic membrane; HAMA; viable human amnion membrane allograft, CAMPs; cellular, acellular, and matrix‑like products.

### 3.2 Healthcare resource costs data

The cost data used in this study was sourced from published literature, see Table 3. The costs included those incurred in the outpatient ambulatory care for healed, unhealed, and infected health states. For amputation, the cost includes that of hospitalization and subsequent care for 1 year. For the intervention costs of treating one DFU, we used a similar approach to that of Samsell et al. 2019^4^ where patients were assumed to be seen in a physician's office. We calculated the mean cost of covering a 5cm^2^ wound as was reported in Samsell paper 2019^4^ by

a) calculating the cost per application.
b) multiply the cost per application by number of applications.

**Table 3:** Cost data and health‐related quality of life (utility) data applied in the model.

Costs of CAMPs					
CAMPs	Cost/sq cm	Wound size	Cost/Application	Applications	Cost of product per treatment
BLCC	$30	44	$1,339	4	$5,355.68
hFDS	$32	37.5	$1,201	4	$4,804.50
vCPM	$134	6	$802	4	$3,209.76
HAMA	$124	6	$747	4	$2,986.08
HSAM	$584	5	$2,918	4	$11,673.40
dHACM	$155	6	$933	4	$3,264.45

**Weekly health state costs**					
**Health state**	**Health state cost**	**Lower value**	**Upper value**	**Source**	
DFU no infection/Unhealed	$673	$538	$808	Rice 2014	
Infected	$349	$279	$418	Hicks^33^	
Amputation	$1,270	$1,016	$1,524	Franklin,^34^ Armstrong^28^	
Healed Ulcer	$0	$0	$0	Assumption	
Death	$0	$0	$0	Assumption	

**Health state utilities**					
**Health State**	**Mean**	**SE**	**Source**		
DFU no infection	0.75	0.15	Redekop^8^		
Healed	0.84	0.17			
Infected	0.70	0.14			
Amputation	0.62	0.12			
Death	0.00	0.00			

vCPM; Cryopreserved placental membrane with viable cells; BLCC, bioengineered bilayered living cellular construct; hFDS, Human fibroblast dermal substitute; dHACM, Dehydrated human amnion chorion membrane; HSAM, hypothermically stored amniotic membrane; HAMA; viable human amnion membrane allograft, CAMPs; cellular, acellular, and matrix‑like products.

DFU, diabetic foot ulcer, SE, standard error.

We used the mean number of applications reported in real‐world clinical studies where applicable and the Centers for Medicare and Medicaid Services (CMS) average selling price per cm^2^ in Q2 2022. For example, we need 2x3cm vCPM to cover a 5cm^2^, and the cost per cm^2^ is $134. The cost per application will be 2x3cm multiplied by the cost per cm^2^ which is ($134 × 6 cm^2^ = $806 per application). We multiplied the cost per application by the mean number of applications reported in the study by Armstrong 2021,^28^ which was 4 for all CAMPs to reflect the real‐world applications. In sensitivity analysis, we used the number of applications that were observed in the clinical studies for each individual CAMP.

The cost of a healed DFU was assumed to be zero in the model, whereas that of an unhealed DFU with no infection which includes the cost of rehabilitation was obtained from a study by Rice et al. 2014.^6^ The study considered deidentified DFU patients with mean ages +65 years and non‐DFU patients with diabetes using Medicare Standard Analytical Files, January 2007–December 2010, and privately insured population. The costs of amputations were taken from a cross‐sectional study by Franklin et al. 2014^34^ while the cost of infection was taken from a retrospective study by Hicks et al. 2016.^33^ All costs were adjusted for inflation using the medical care inflation to 2021 prices according to the US Bureau of Labour Statistics (https://www.in2013dollars.com/Medical-care/price-inflation). The time horizon or the analysis period was 52 weeks, therefore we did not discount the costs or the benefits according to good practice guidelines of economic evaluations.^23^, ^24^ In a scenario analysis, we evaluated the model at 12 and 26 weeks to see if the time horizon would impact the model conclusions.

### 3.3 Health‐related quality of life (HRQOL)

Data on healed wounds and quality‐adjusted life years (QALYs) were considered as outcomes for this analysis. QALYs were estimated by multiplying the utility data and the time spent in a health state. The utility data was obtained from the literature.^8^ Redekop et al. 2004^8^ used the generic EuroQol instrument to evaluate health‐related quality of life and generated the utilities of DFU and its complications using the time‐trade‐off method.

### 3.4 Cost‐effectiveness analysis and sensitivity analysis

The incremental cost‐effectiveness ratio (ICER) was calculated as the difference in costs minus the difference in QALYs or healed wounds. A willingness to pay threshold of $50‐100,000/QALY and per healed wound to determine if an intervention was cost‐effective or not in accordance with guidance from the ICER Institute.^35^

Our base case model first compared the cost‐effectiveness of all CAMPs with SoC over 52 weeks. We then indirectly compared CAMPs against each other using SoC results as the basis for comparison by subtracting the total cost and benefits of SoC from each CAMP (making SoC the origin of the decision plane). When more than two interventions are being compared, the ICERs are calculated using the following process:

The CAMPs are ranked in terms of cost, from the cheapest (total treatment costs) to the most expensive, and exclude dominated options (the CAMP is more expensive and less effective compared to the previous one). ICERs are then calculated for each CAMP compared with the next most expensive nondominated CAMP. If the ICER for a CAMP is higher than that of the next most effective CAMP, then it is ruled out by ‘extended dominance’. The final ICERs reported are recalculated, excluding any CAMPs subject to dominance or extended dominance.

Model uncertainty was assessed using one‐way sensitivity analysis, that is varying data inputs one at a time and evaluating the ICER. Key parameters that were varied included rates of healing, infections, amputations, and costs using data obtained from published literature like the 95% confidence intervals, where there was no variation data provided, we varied the inputs by plus or minus 20%. To assess the simultaneous impact of changing all the parameters at once, we implemented a probabilistic sensitivity analysis (PSA) by assigning probability distributions to the model inputs. We assigned a gamma and beta distribution for cost and utility respectively, while for clinical effectiveness parameters (healing rates) we assigned the log‐normal distribution. We conducted 5,000 simulations to simulate the costs and outcomes of the different comparators. To visualize the uncertainty from the PSA, we present cost‐effectiveness acceptability curves.

## 4 RESULTS

**Figure 2:**
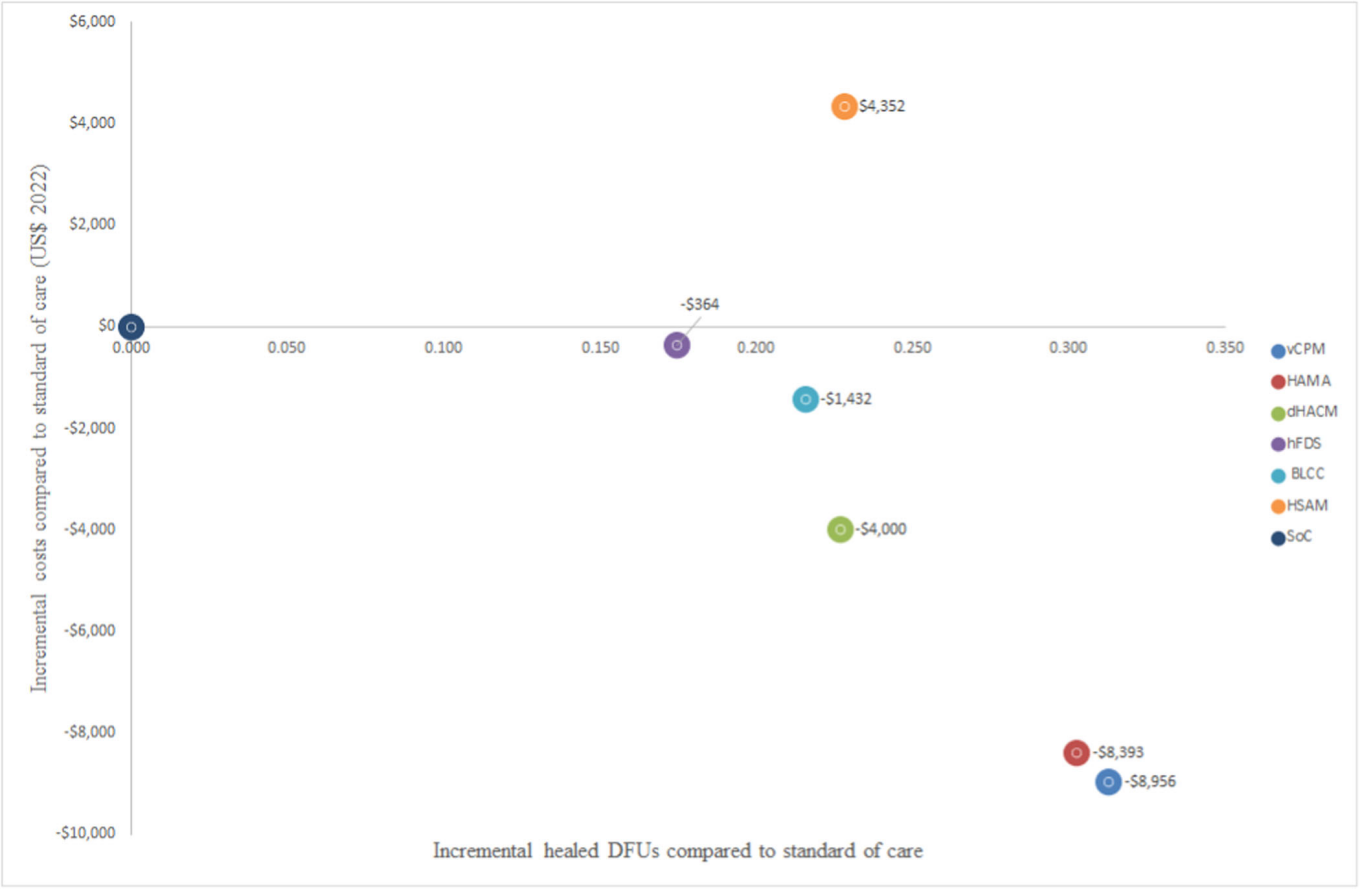
Scatter plot showing the cost‐effectiveness results using healed DFUs as the outcome measure.

**Figure 3:**
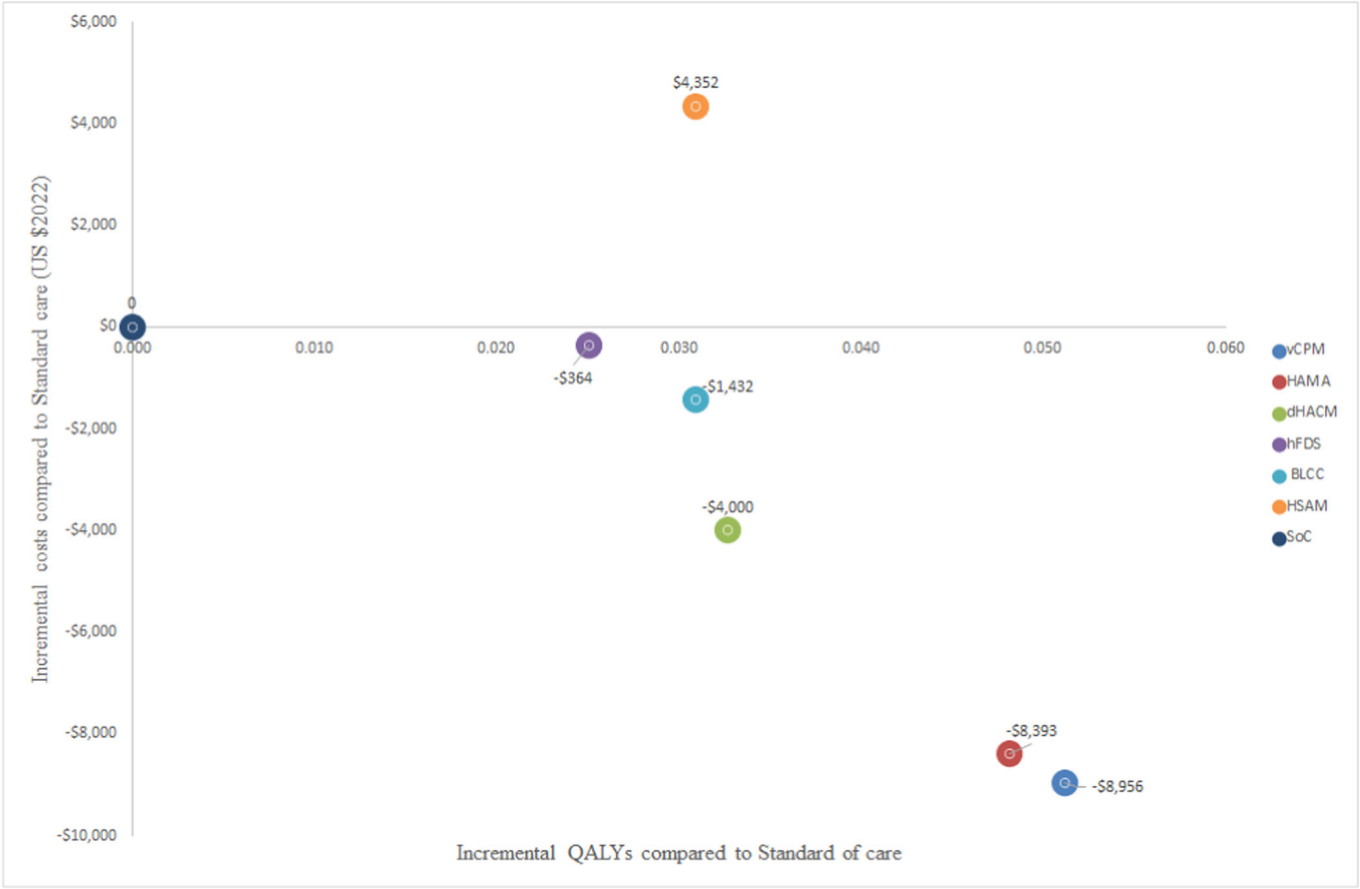
Scatter plot showing the cost‐effectiveness results using QALYs as the outcome measure.

The CAMPs were all found to be cost‐saving when compared with SoC at 1 year, that is they were cheaper overall and were more effective (more healed wounds and quality‐adjusted life years). The only CAMP that was not cost‐saving compared to SoC was HSAM which was nonetheless cost‐effective, it resulted in incremental costs and benefits. HSAM has an estimated ICER OF $19,077/healed DFU or $139,480/QALY compared to SoC. The analysis also showed that among the CAMPs, vCPM is the most cost‐effective, dominating other CAMPs over 1 year when they are compared indirectly via SoC as the baseline (Table 4 and Table 5 and Figures 2 and 3).

**Table 4:** Results: Mean costs and outcomes per treated patient for all CAMPs and SoC.

Intervention	Cost ($)	Effect (QALYs)	Effect (closed wounds)
vCPM	$10,907	0.783	0.914
HAMA	$11,470	0.780	0.903
dHACM	$15,862	0.764	0.828
hFDS	$18,430	0.763	0.816
BLCC	$19,498	0.757	0.775
SoC	$19,862	0.732	0.601
HSAM	$24,214	0.763	0.829

vCPM; Cryopreserved placental membrane with viable cells; BLCC, bioengineered bilayered living cellular construct; hFDS, Human fibroblast dermal substitute; dHACM, Dehydrated human amnion chorion membrane; HSAM, hypothermically stored amniotic membrane; HAMA; viable human amnion membrane allograft, QALYs‐quality‐adjusted life years.

**Table 5:** Results: Ranked cost‐effectiveness of CAMPs using SoC as the baseline anchor showing the cost saving and outcomes patient.

Intervention	Costs compared to SoC	Effects Wound healed vs SoC	Effects QALYs vs SoC	Cost/healed DFU	Cost/QALY
SoC	0	0	0		
vCPM	‐$8,956	0.313	0.051		
HAMA	‐$8,393	0.302	0.048		
dHACM	‐$4,000	0.227	0.033		
hFDS	‐$364	0.174	0.025		
BLCC	‐$1,432	0.216	0.031		
HSAM	$4,352	0.228	0.031	$19,077	$139,480

SoC‐standard of care, QALYs‐ quality‐adjusted life years, vCPM; Cryopreserved placental membrane with viable cells; BLCC, bioengineered bilayered living cellular construct; hFDS, Human fibroblast dermal substitute; dHACM, Dehydrated human amnion chorion membrane; HSAM, hypothermically stored amniotic membrane; HAMA; viable human amnion membrane allograft.

## 5 SENSITIVITY ANALYSIS

We conducted a one‐way sensitivity analysis and the results demonstrated that the model was sensitive to the relative risk of wound closure for the CAMPs compared to SoC and the number of CAMP applications over the treatment period. The model assumed that most of the CAMP have 4 applications as was reported in a real‐world evidence study by Armstrong who found a mean application of 3.7. When we changed the assumption around number of applications from 4 to those observed in the clinical studies, 5 for BLCC, 6 for vCPM, 7.1 for hFDS, and 3.5 for dHACM, while HAMA and HSAM were as modeled in the base case, the results changed slightly. Conclusions remained that vCPM was still the cost‐effective CAMP; however, it was no longer dominant as before. Similar results were seen when the treatment effect was varied suggesting that the model is slightly sensitive to assumptions around the number of applications between CAMPs. Table 6 shows the results per wound closed when the number of applications is as reported in the clinical studies. When other variables were changed, such as age, utility, mortality, and the cost of the health states, vCPM remained the dominant choice.

**Table 6:** Sensitivity analysis when CAMPs applications are as reported in clinical studies.

Intervention	Cost ($)	Effect (closed wounds)	Simple dominance?	Excluding simple dominance		
				Incremental cost ($)	Incremental effect	ICER ($/closed wound)
HAMA	$11,470	0.903				
vCPM	$12,511	0.914	N	$1,042	0.0105	$99,657
dHACM	$15,862	0.828	Y	‐	‐	‐
BLCC	$19,769	0.816	Y	‐	‐	‐
SoC	$19,862	0.601	Y	‐	‐	‐
hFDS	$23,222	0.775	Y	‐	‐	‐
HSAM	$24,214	0.829	Y	‐	‐	‐

vCPM; Cryopreserved placental membrane with viable cells; BLCC, bioengineered bilayered living cellular construct; hFDS, Human fibroblast dermal substitute; dHACM, Dehydrated human amnion chorion membrane; HSAM, hypothermically stored amniotic membrane; HAMA; viable human amnion membrane allograft.

### 5.1 Scenario analysis

For the scenario analyses, we focused on the impact of the follow‐up period on model conclusions, that is we assessed the results at 12 weeks and 26 weeks. This analysis showed that all CAMPs are cost‐effective or cost‐saving when compared to SoC. This also showed that over 12 weeks, vCPM will be the most cost‐effective CAMP compared to the next best choice HAMA with an estimated ICER of $90/healed DFU or $1,269/QALY while it dominates all the other CAMPs. At 26 weeks the results are cost‐saving in favor of vCPM compared to either SoC or other CAMPs.

### 5.2 Probabilistic sensitivity analysis

**Figure 4:**
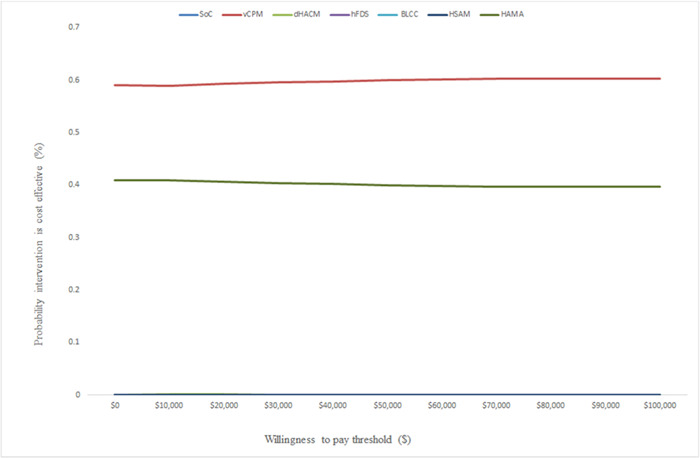
Cost‐effectiveness acceptability curves showing the probability that each intervention is cost‐effective using healed wounds as a measure of outcome.

**Figure 5:**
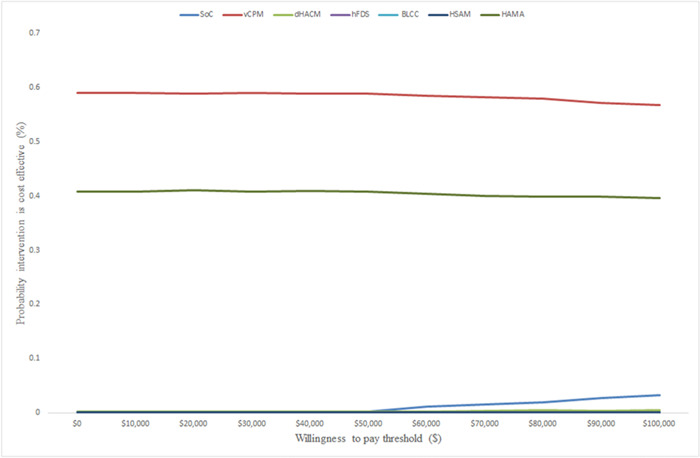
Cost‐effectiveness acceptability curves showing the probability that each intervention is cost‐effective using QALYs as a measure of outcome.

Figures 4 and 5 show the results of the PSA over 52 weeks. These results show that vCPM has a 60% probability of being cost‐effective when compared to the rest of the CAMPs followed by HAMA at 40% at $100,000/healed wound. When QALYs are used as the measure of outcome, the same findings were seen and vCPM had a 59% probability of being cost‐effective followed by HAMA with a 40% probability of being cost‐effective. The probabilistic results confirmed the deterministic results and therefore given the current evidence, we can be confident that vCPM is the most cost‐effective CAMP followed by HAMA, dHACM, hFDS, BLCC, and lastly HSAM.

## 6 DISCUSSION

Several systematic reviews and meta‐analyses Su et al. 2020^13^ (7 studies), Paggiaro et al. 2018^14^ (6 studies), Guo et al. 2017^15^ (6 studies), and Haugh et al. 2017^16^ (5 studies) found statistically significant evidence in favor of CAMPs compared to SoC in treating DFUs. This was particularly the case with regard to healing and time to healing. Additionally, observational studies of CAMPs have corroborated evidence from RCTs. For instance, Raspovic et al. 2018^36^ using real‐world data from electronic health records found that 59.4% of wounds treated with vCPM achieved wound closure. Another observational study using NetHealth electronic medical records compared BLCC with or dHACM and found healing rates at 12 weeks of 48% for BLCC and 24% for dHACM.^37^ Martinson et al. 2016^38^ used the CMS claims data to assess the use of Urinary Bladder matrix (UBM); BLCC; hFDS; and tri‐layer porcine small intestine submucosa (SIS; Oasis Ultra) in DFU. They found 13193 skin episodes treatment and found that at 12 weeks the percentage of wounds that healed were UBM 62%; SIS 63%; BLCC 58%; and hFDS 58%.

Earlier economic evaluations found that the use of CAMPs as an adjunct to SoC are cost cost‐saving when compared with SoC alone. A recent US study by Tettelbach et al. 2022^20^ found that the use of advanced treatment DHACM was cost‐saving compared to non‐advanced treatment. It resulted in improved quality‐adjusted years of 0.013 and saving $3,670 per patient. These results are similar to our findings where we estimated that DHACM will save $4,000 and improve QALYs by 0.033 for the same comparison. One study in Switzerland compared BLCC with SoC in patients with DFU over 1 year.^8^ They concluded that BLCC was cost‐saving as it resulted in lower costs overall and improved healing outcomes. In a study comparing SIS with SoC, Guest et al. 2017,^7^ found SIS to be cost‐saving in the management of DFU patients. Another study performed in France in 2000 showed that hFDS had a lower average cost of treatment compared to SoC because of improved outcomes in DFU patients.^19^ Our analysis differs from these because it is based on the most recent data and includes more recent CAMPs. In addition, our analysis compared the CAMPs indirectly against each other using SoC as a common comparator. This is an indirect way of comparing treatments in the absence of direct evidence. We were able to rank the CAMPs in order of cost‐effectiveness using Monte Carlo simulation by calculating the probability that each intervention had the highest net health benefits.

A clear result from this analysis is that using CAMPs as an adjunct to SoC is cost‐effective compared to SoC alone. This result is driven by the effectiveness of CAMPs reported in many systematic reviews and meta‐analyses as pointed out earlier. Our analysis showed the savings range from $0.4 m to $8.96 million per year for 1000 treated patients. In addition, the analysis also showed that among the CAMPs, vCPM is the most cost‐effective with the highest probability of having the highest net health benefit of 60%, followed by HAMA at 40%, HAMA, dHACM, hFDS, BLCC and lastly HSAM using the current evidence.

At 1 year vCPM was found to dominate the other CAMPs. This was driven by higher healing rates seen when compared to standard of care. vCPM was no longer dominant compared to SoC when the follow‐up period was reduced to 12 weeks. It was now estimated to be cost‐effective with a cost per healed wound of $90 and $1,269/QALY compared to HAMA. Furthermore, the uncertainty of model findings due to individual parameters was assessed via one‐way and probabilistic sensitivity analysis. The results were sensitive to changes in the risk ratio of wound healing and the number of applications of the CAMPs. Probabilistic sensitivity analysis confirmed that there is a degree of uncertainty in the base case results since vCPM had a 60% probability of yielding the greatest net health benefits followed by HAMA with 40%, which is the next best alternative.

## 7 LIMITATIONS

This evaluation has some limitations as is the case with most economic evaluations. Firstly, effectiveness data was taken from RCTs which gives us information about the efficacy of the CAMPs rather than their effectiveness. However, we noted that for those CAMPs that had evidence from real‐world settings, the findings were not significantly different from RCT results. Second, we did not have a direct head‐to‐head comparison of the CAMPs and therefore conducted the analysis using an indirect approach. All CAMPs assessed had evidence against SoC, and we used this to make an indirect comparison between them. This method is acceptable in the absence of direct evidence; however, the analysis would need updating when direct evidence becomes available.

The analysis assumed that there would be no difference between CAMPs regarding outcomes of amputation and infections. This may not necessarily be true as there is evidence supporting that the risk of infection and amputation is associated with unhealed wounds. Seeing that these CAMPs have varying degrees of healing rates, this may bias the results of the analysis against those CAMPs that have better healing rates. Furthermore, data on amputation was taken from a real‐world evidence study by Armstrong et al. 2021,^28^ we acknowledge this data source contains mixed patients, which may not be reflective of patients assessed in the different clinical trials. We recommend that data on amputation should be collected in future comparative studies of CAMPs. One of the problems of meta‐analysis is heterogeneity both clinical and statistical. Statistical heterogeneity was mitigated by using the random effects model, which weights the mean effects inversely to total study variation.^39^ For clinical heterogeneity, we acknowledge that although studies were not entirely homogeneous, most of the baseline parameters were similar.

Our study used cost data and average selling prices for the CAMPs from the US; therefore, these results apply to the US healthcare systems, other healthcare jurisdictions are encouraged to use their local applicable costs. The base case model assumed 4 applications of CAMP per wound obtained from a large, published study,^28^ we acknowledge that the mean number of applications may vary from patient to patient and also depends on commercial payers who may impose limits on the number of applications. Indeed, we changed this assumption in sensitivity analysis and the results were slightly sensitive to changes in this assumption. Finally, this study was conducted from a payer perspective; we suggest evaluating future studies from other perspectives, such as a hospital perspective.

Overall, all CAMPs were shown to be cost‐effective when compared to SoC alone in managing DFUs. However, vCPM appears to be the most cost‐effective CAMP over the modeled 52 weeks followed by HAMA, dHACM, hFDS, BLCC, and HSAM. We urge caution in interpreting the results because we currently lack head‐to‐head evidence comparing all these CAMPs and therefore suggest that this analysis should be updated when more direct evidence of CAMPs becomes available.

All authors have read and approved the final version of the manuscript, Leo Nherera had full access to all of the data in this study and takes complete responsibility for the integrity of the data and the accuracy of the data analysis.

## AUTHOR CONTRIBUTIONS

**Leo M Nherera**: Conceptualization; Data curation; Formal analysis; Methodology; Project administration; Writing—original draft; Writing—review & editing. **Jaideep Banerjee**: Conceptualization; Data curation; Validation; Writing—review & editing.

## CONFLICT OF INTEREST STATEMENT

LN and JB are employees of Smith + Nephew Inc. and may own shares of Smith + Nephew.

## ETHICS STATEMENT

The authors have nothing to report.

## TRANSPARENCY STATEMENT

The lead author Leo M. Nherera affirms that this manuscript is an honest, accurate, and transparent account of the study being reported; that no important aspects of the study have been omitted; and that any discrepancies from the study as planned (and, if relevant, registered) have been explained.

## Data Availability

The data supporting the findings of this study are available within the article and additional information is available upon request from the corresponding author. The data that support the findings of this study are available from the corresponding author upon reasonable request.
